# Direct surface coating of high voltage LiCoO_2_ cathode with P(VDF-HFP) based gel polymer electrolyte†

**DOI:** 10.1039/d0ra04023a

**Published:** 2020-06-26

**Authors:** Huiling Chen, Yuehua Wen, Yue Wang, Songtong Zhang, Pengcheng Zhao, Hai Ming, Gaoping Cao, Jingyi Qiu

**Affiliations:** Beijing Key Laboratory of Advanced Chemical Energy Storage Technology and Materials, Research Institute of Chemical Defense Beijing 100191 China wen_yuehua@126.com qiujingyi1202@163.com

## Abstract

For high-voltage cycling of lithium-ion batteries, a gel polymer Li-ion conductor layer, P(VDF-HFP)/LiTFSI (PHL) with high electrochemical stability has been coated on the surfaces of as-formed LiCoO_2_ (LCO) cathodes by a solution-casting technique at low temperature. An LCO cathode coated with around 3 μm thickness of the PHL ultrathin membrane, retains 88.4% of its original capacity (184.3 mA h g^−1^) after 200 cycles in the 3.0–4.6 V range with a standard carbonate electrolyte, while the non-coated one retains only 80.4% of its original capacity (171.5 mA h g^−1^). The reason for the better electrochemical behaviors and high-voltage cycling is related to the distinctive characteristics of the PHL coating layer that is compact, has highly-continuous surface coverage and penetrates the bulk of LCO, forming an integrated electrode. The PHL coating layer plays the role of an ion-conductive protection barrier to inhibit side reactions between the charged LCO surface and electrolyte, reduces the dissolution of cobalt ions and maintains the structural stability of LCO. Further, the PHL coated LCO cathode is well preserved, compared to the uncoated one which is severely cracked after 200 cycles at a charging cut-off voltage of 4.6 V.

## Abstract 2

For high-voltage cycling of lithium-ion batteries, a Li-ion conductor layer, P(VDF-HFP)/LiTFSI with high electrochemical stability has been coated on the surfaces of as-formed LiCoO_2_ cathodes by a solution casting technique at low temperature.

## Graphical abstract

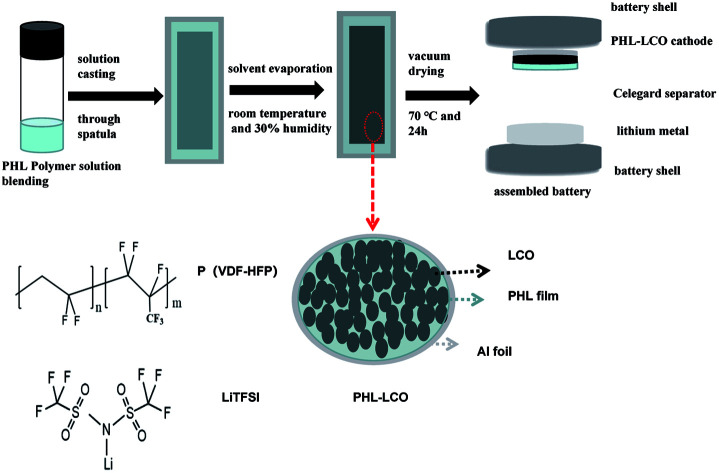

## 1. Introduction

For the application of high energy-density lithium-ion batteries (LIBs), it is extremely attractive to research and develop cathode materials with large reversible capacity and high operating voltage.^1–5^ Unlike high power-density materials such as LiNi_1/3_Co_1/3_Mn_1/3_O_2_,^6,7^ lithium cobalt oxide (LCO) still occupies a dominant position in the cathode materials of LIBs, because its advantages of high volumetric energy-density, facile fabrication process and well-balanced electrochemical performance. However, the current discharge capacity of LCO with a general charging cut-off voltage of 4.2 V is only 140 mA h g^−1^, although its theoretical capacity is 274 mA h g^−1^.^8^ One effective attempt to improve the average voltage and discharge capacity of LCO is to increase the charging cut-off voltage to more than 4.2 V.^9–12^ But when the charging cut-off voltage is enhanced to over 4.2 V, particularly at 4.5–5 V, the LCO battery suffers from increased surface side reactions, large reaction polarization and structural deterioration.^13,14^ It is confirmed that these irreversible changes mainly occur on the surface of LCO.^15–17^ Therefore, surface modification of LCO by various materials combined with metal doping has become a recognized and effective method. Inorganic materials involve metal oxide materials and solid electrolyte such as Mg doping and ZrO_*x*_F_*y*_ coating,^18^ Al_2_O_3_,^19^ La_2_O_3_,^20^ AlPO_4_,^21^ ZrO_*x*_F_*y*_,^22^ Li_2_MnO_3_,^23^ Li_1+*x*_Al_*x*_Ti_2−*x*_(PO_4_)_3_ (LATP)^12^ and Lipon^24^*etc.* Among these, metal oxides and inorganic solid electrolytes usually are discontinuously deposited onto LCO. The metal oxide coating could hinder lithium-ion transport between the cathode and electrolyte, resulting in high interfacial resistance. While, the ion conductivity of LATP and Lipon is only 10^−6^ S cm^−1^ at 20 °C and the tolerance for 4.5–5 V high voltage excursions is discounted. Comparatively, only a few organic materials are used. To solve the previous problems of traditional inorganic material coatings, J. Park *et al.* exploited the polyimide (PI) gel polymer electrolyte (GPE) coating of LCO,^25^ which is a lithium-ion conducting coating layer. However, the high voltage cycling performance of LCO is seriously limited (only ∼50 cycles), maybe attributed to the insufficient resistance for high voltage oxidation. The cycling performance of the surface modified LCO by inorganic and organic materials at 4.6 V charge cut-off voltage is summarized in the following Table 1. It shows that the capacity retention rate of the modified LCO at 1C is poor. Otherwise, the cycle number is rather few.

**Table 1:** The cycling performance of coated LCO used for LIBs at the 4.6 V charge cut-off voltage

Cathode material and reference	Cutoff voltage and cycling rate	Initial discharge capacity (mA h g^−1^)	Capacity retention ratio
LCO@Al_2_O_3_ (ref. 19)	3.0–4.6 V, 0.5C	204.4	55% (after 80 cycles)
LCO@La_2_O_3_ (ref. 20)	2.75–4.6 V, 0.2C	195.0	83% (after 60 cycles)
LCO@AlPO_4_ (ref. 21)	3.0–4.6 V, 1C	179.5	82% (after 46 cycles)
LCO@ZrO_*x*_F_*y*_ (ref. 22)	3.0–4.6 V, 1C	195.6	33% (after 200 cycles)
LCO@Li_2_MnO_3_ (ref. 23)	3.0–4.6 V, 1C	195.4	52% (after 100 cycles)
LCO@PI (ref. 25)	3.0–4.6 V, 0.5C	198.5	64% (after 50 cycles)
This work	3.0–4.6 V, 1C	184.3	88.4% (after 200 cycles)

**Fig. 1:**
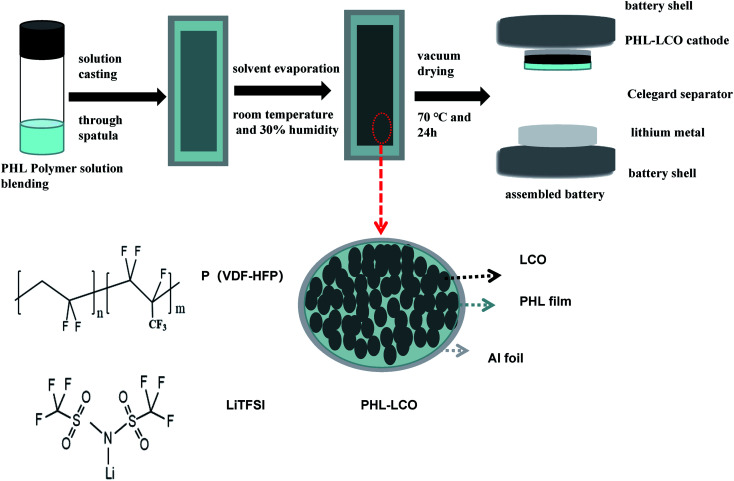
The preparation process of PHL coated LCO cathodes.

Here, the P(VDF-HFP)/LiTFSI (PHL) GPE possesses high electrochemical stability and was introduced on LCO surface through a facile solution casting and solvent evaporation route as shown in Fig. 1. Phase inversion reaction of PHL GPE is conducted directly on as-formed LCO cathode, instead of application to LCO powders. Thereby, it would not damage preformed physical architecture of the modified LCO electrode (specifically, electronic conductive networks). Following, based on morphological and structural characterization of the PHL layer-coated LCO cathode, advantageous effects of the PHL coating layer on cycling performance and structural stability of 4.6 V high-voltage LCO are investigated. The results show that the effective ion conductive barrier coating significantly enhances the cycling performance and structural stability of the LCO at high cut-off voltage of 4.6 V.

## 2. Experimental

### 2.1 Materials

Polyvinylidenefluoride-hexafluoropropylene (P(VDF-HFP), Mn = 400 000, 99.9%) and lithium bis(trifluoromethansulfon)imide (LiTFSI, 99.9%) were obtained from Aladdin Company. Commercial lithium cobaltate (LCO, 99.9%) was purchased from Contemporary Amperex Technology Co. Ltd. Other chemicals were analytical-grade reagents obtained from Beijing Chemical Company.

### 2.2 Synthesis of PHL coated LCO cathodes

P(VDF-HFP) and LiTFSI were dissolved in N–N dimethyl formamide (DMF) (1.5 : 0.1 : 5 by weight) and mixed uniformly at 20 °C for 24 h. Next the mixture was poured into a Al foil to evaporate the solvent and dried in vacuum at 70 °C for 48 h. Further, the polymer membranes were swelled in liquid electrolyte (LE) for 6 h in a glovebox. And the obtained GPE is abbreviated as PHL GPE.

The LCO cathode was prepared by mixing 90% LCO powder, 5% carbon black and 5% PVDF binder in proportion. Surface modified LCO cathode was prepared by the solvent-casting method.^26–28^ LCO cathode was cast with pre-prepared polymer solution by a doctor blade with the gap of 40 μm, 50 μm, 60 μm and 80 μm, respectively. Then slurry-coated LCO cathode dried in vacuum at 70 °C. The actual thickness of the PHL polymer film on the surface of modified LCO cathodes is 1 μm, 3 μm, 5 μm, 8 μm, respectively. The corresponding LCO cathodes are recorded as 1-PHL-LCO, 3-PHL-LCO, 5-PHL-LCO, 8-PHL-LCO.

### 2.3 Characterization

The morphology and element distribution of the PHL LCO cathodes were obtained by a SU8010 scanning electron microscope (SEM) with energy spectrometer (EDAX). The structure of cathode materials was observed by a D8 Advance X-ray diffraction (XRD) with Cu-Kα radiation (*λ* = 1.5406 Å) in the range of 10–80° and at a speed of 5° min^−1^. The content of Co^3+^ in the electrolyte after half batteries cycling was measured by S4 T·STAR X-ray Fluorescence Spectrometer (XRF).

Linear sweep voltammetry (LSV) was used to evaluate the electrochemical stability of PHL GPE. The PHL film was sandwiched between lithium electrode and stainless steel electrode with a scanning speed of 0.1 mV s^−1^. The AC impedance of PHL GPE and modified LCO cathodes was measured on an impedance analyzer (PMC2000 1000 500, America) with a frequency of 100 kHz to 0.1 Hz and a disturbance amplitude of 5 mV. The unit cell (type 2025 coin) was assembled by a separator sandwiched between a PHL-coated LCO cathode and a lithium metal anode, and then filled with 30 μL LE. The cycling property of the cells was precycled at 0.1C for 3 cycles, and then cycled at 1C (1C = 220 mA h g^−1^). The rate capability was tested under different current densities. All half-cells were impregnated on LANHE CT2001A for 6 h, and then tested between 3.0 and 4.6 V.

## 3. Results and discussion

### 3.1 Characterization of PHL GPE

**Fig. 2:**
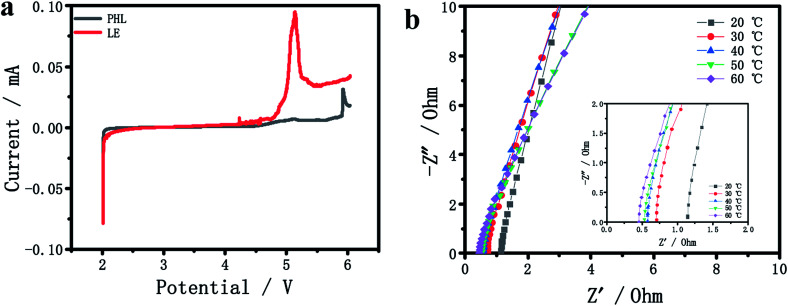
(a) LSV curves of PHL GPE and LE. (b) Impedance plots of the PHL GPE at different temperatures.

The electrochemical stability window of PHL GPE and LE at room temperature was compared by LSV. In Fig. 2a, there is no obvious decomposition current occurs in PHL GPE up to 5 V, while LE decomposes at about 4.3 V. The results show that PHL GPE has favorable electrochemical stability, which indicates that PHL GPE is a promising polymer electrolyte for higher voltage LIBs.

Ionic conductivity is the most significant parameter for studying the electrochemical performance of electrolyte membrane.^29–34^ The bulk resistance (*R*_b_) of the PHL GPE was measured by AC impedance spectrometer, and the conductivity was calculated according to the literature,^35,36^Fig. 2b shows the *R*_b_ at different temperature from 20 °C to 60 °C. Obviously, the value of *R*_b_ becomes smaller with the increasing temperature corresponding to the increasing ionic conductivity. The low *R*_b_ value of PHL GPE at 20 °C is 1.1 Ω corresponding to the ionic conductivity 6.5 × 10^−3^ S cm^−1^, which may be attributed to the high ionic conductivity network of PHL GPE.

### 3.2 Characterization of integrated PHL coated LCO

**Fig. 3:**
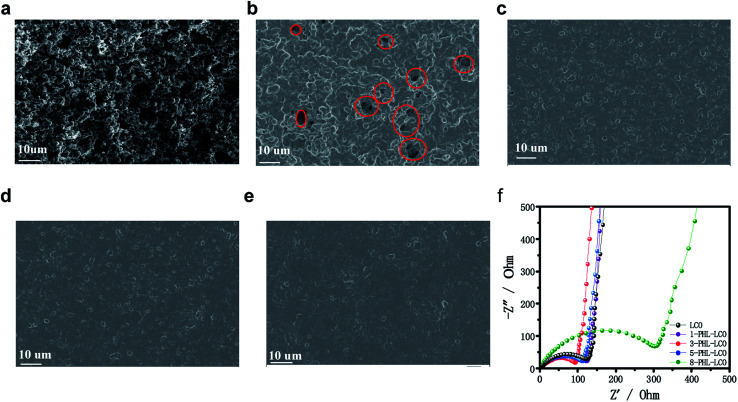
SEM images of (a) uncoated LCO, (b) 1-PHL-LCO, (c) 3-PHL-LCO, (d) 5-PHL-LCO and (e) 8-PHL-LCO. (f) Impedance plots of uncoated and coated LCO half-cells.

The surface morphology of coated LCO with different thickness and uncoated one is shown in Fig. 3. Obviously, the GPE layer of PHL coating prepared by solvent casting method successfully covers onto the surface of LCO cathode, forming different integrated LCO electrodes. Among the surface modified LCO cathodes, the surface of 1-PHL-LCO cathode shows relatively rough surface with some holes distributed on the LCO cathode surface, which is associated with the extremely thin GPE membrane. As the thickness of the PHL coating layer increases, the surface coverage increases. When the thickness exceeds 3 μm, a uniform and relatively dense membrane-like coating is formed.

In order to optimize the thickness of PHL coated LCO cathodes, the AC impedance spectra of half-cells for the PHL coated LCO cathodes with different thickness was analyzed. It has been reported that the semicircle of at the high frequency range in impedance spectra belongs to the Li-transferring interfacial resistance (*R*_int_) of the surface membrane on the electrodes. In general, a membrane with low *R*_int_ and high ionic conductivity is beneficial to enhance the electrochemical performance of electrodes. As shown in Fig. 3f, the *R*_int_ of 3-PHL-LCO cathode is the smallest, which may be the result of the combined effect of surface morphology and thickness of the film.

**Fig. 4:**
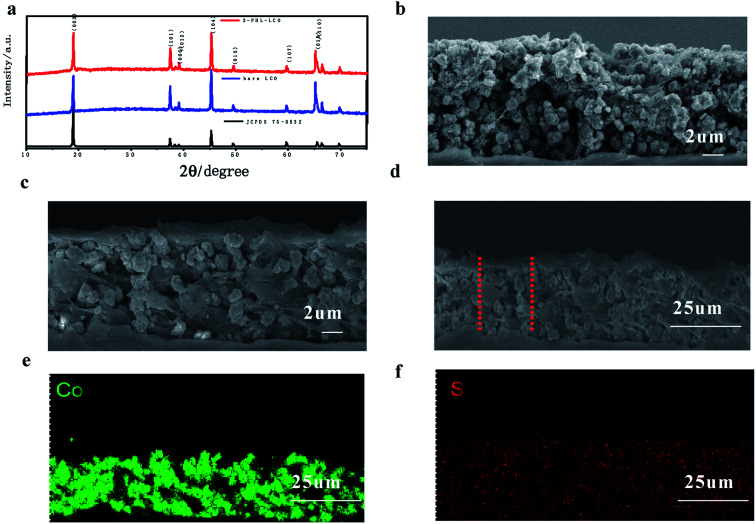
(a) XRD patterns of LCO and 3-PHL-LCO. SEM image cross sections of (b) LCO and (c) 3-PHL-LCO. (d) SEM image and the corresponding (e) Co and (f) S of 3-PHL-LCO.

The XRD was used to contrast the structure of PHL coated LCO cathode and pristine one. Fig. 4a shows that both samples have a hexagonal crystal structure, belonging to *R*-3*m* space group and no impurity peaks are detected in 3-PHL-LCO, indicating that the crystal structure of LCO remains unchanged after the coating of PHL. Fig. 4 gives the SEM images of the cross section of 3-PHL-LCO cathodes. Compared with the original porous and loose morphology, the cross section of a 3-PHL-LCO cathode also appears more smooth and dense. The whole cross section of the coated LCO cathode is determined as the zone of Co and S elemental mappings, where Co and S represent the distribution of LCO and PHL coating layer, respectively. The comparison of Fig. 4e and f indicates that S component almost completely penetrates into the internal bulk of the electrode. This leads to the above densification in the modified LCO cathode. An increase in the electrode thickness also suggests that an ultrathin film is encapsulated on the surface area of the sample. Overall, the PHL coated LCO cathode has been integrated with this polymer electrolyte membrane.

### 3.3 Electrochemical characterization

**Fig. 5:**
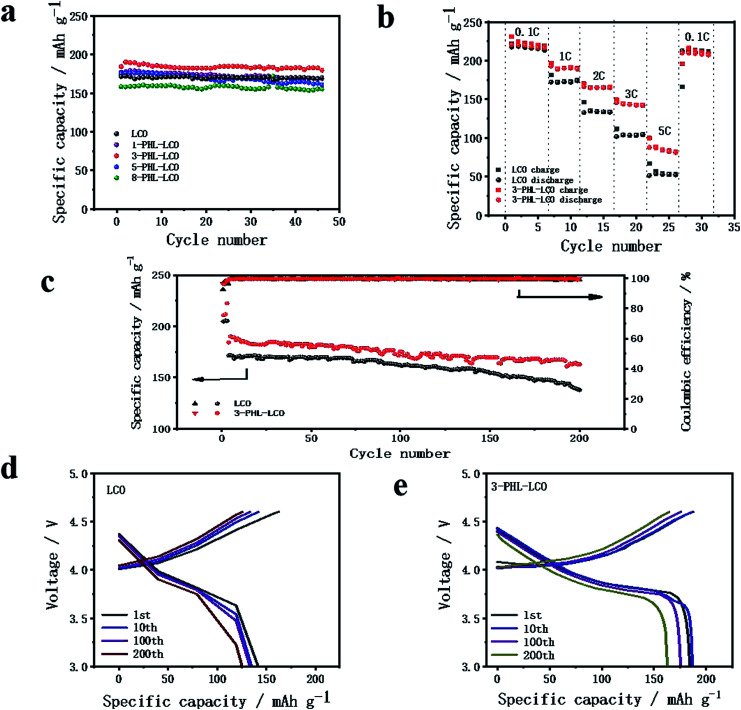
(a) Cycle performance of LCO and PHL coated LCO cathodes at 1 C-rate. (b) Rate performance under different current densities and (c) long cycle performance of bare LCO and 3-PHL-LCO. Variation of charge–discharge curves at 1st, 10th, 100th, 200th cycle of (d) bare LCO and (e) 3-PHL-LCO.

The influence of PHL coating on the electrochemical performance of LCO was evaluated by charge–discharge measurement. The modified LCO cathodes with different thickness of PHL coating were tested between 3.0 and 4.6 V at 1C rate (220 mA h g^−1^). The cycle performance is exhibited in Fig. 5a, except for 5-PHL-LCO, the PHL coated LCO and bare LCO show excellent electrochemical stability and the discharge capacity retention is around 97% after 46 cycles. However, the initial discharge capacity is significantly different, which is 176.9 mA h g^−1^, 184.3 mA h g^−1^, 175.2 mA h g^−1^ and 158.4 mA h g^−1^ for the cathode with the PHL electrolyte membrane in thickness of 1 μm, 3 μm, 5 μm and 8 μm, respectively. For thickness range of PHL coating from 1 μm to 5 μm, the modified LCO do not compromise the capacity compared to the bare LCO (171.5 mA h g^−1^). But the initial discharge capacity decreases with the further increase of the thickness. In general, the 3-PHL-LCO presents excellent cycling stability and the highest discharge capacity. Fig. 5b shows the rate performance of 3-PHL-LCO and bare LCO at different current densities in the 3.0–4.6 V range. Obviously, the discharge capacity of bare LCO is only 51.4 mA h g^−1^ at 5C, while 3-PHL-LCO can still provide 87.8 mA h g^−1^. Furthermore, the 3-PHL-LCO can still deliver a high reversible capacity of 210 mA h g^−1^ at 0.1C, whereas the reversible capacity of bare LCO is 166.1 mA h g^−1^ after cycling at 5C rate, demonstrating the high stability of the modified LCO during high-rate cycling. The long-term cycling performance of 3-PHL-LCO at 1C is shown in Fig. 5c. Compared to the pristine LCO, the 3-PHL-LCO exhibits significantly improved capacity and cycle performance. The initial discharge capacity of pristine LCO and 3-PHL-LCO is 171.5 mA h g^−1^ and 184.3 mA h g^−1^, respectively. In the first 80 cycles, there is almost no difference between the original LCO and 3-PHL-LCO. However, in the next cycle, discharge capacity of 3-PHL-LCO declines extremely retarded as the number of cycles increases, while the capacity of the original LCO continues to decline. After 200 cycles, the capacity retention rate of 3-PHL-LCO is as high as 88.4%, while that of the original LCO drops to 80.4%. In addition, the first coulombic efficiency of bare LCO is only 92.74%, but that of 3-PHL-LCO is 96.40%. And 3-PHL-LCO always shows higher coulombic efficiency, compared with uncoated LCO. It indicates that the PHL coating could reduce the side reactions on surface of LCO. In order to understand the effect of PHL coating on LCO, the selected charge–discharge curves of bare LCO and 3-PHL-LCO at 1st, 10th, 100th, 200th cycle are compared. According to Fig. 5d and e, it can be seen clearly that the 3-PHL-LCO with well maintained capacity just exhibits a small increase in polarization, but the reaction polarization of original LCO increases significantly and thus the capacity rapidly fades with the increase of cycle numbers, which is consistent with the result of d*Q*/d*V* curves (Fig. S1†).

**Fig. 6:**
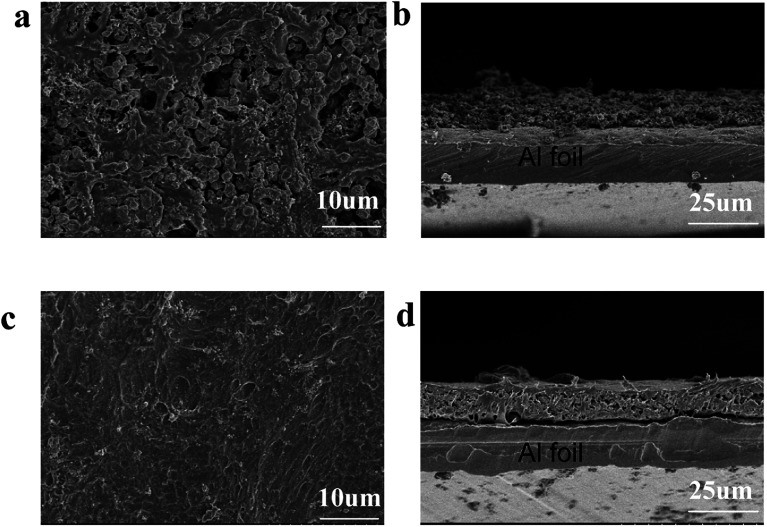
SEM images of cathodes after 200 cycles. Bare LCO (a) surface and (b) cross section, 3-PHL-LCO (c) surface and (d) cross section.

Fig. 6 shows the SEM images of 3-PHL-LCO and bare LCO at full discharge state with the cutoff potential of 3.0–4.6 V after 200 cycles at 1 C-rate. With repeated cycling, the surface of bare LCO electrode is severely cracked, while that of the PHL-coated cathode is still highly-continuous and dense. The cross-section image of uncoated LCO displays a large number of visible particles distributed on the surface, which is easy to fall off from the current collector resulting in a significant increase of the cathode resistance. In contrast, this PHL electrolyte membrane can be well preserved at 4.6 V, indicating great contact between PHL electrolyte membrane and LCO cathode sheet and good structural stability of the cathode sheet during the long cycling process.

**Fig. 7:**
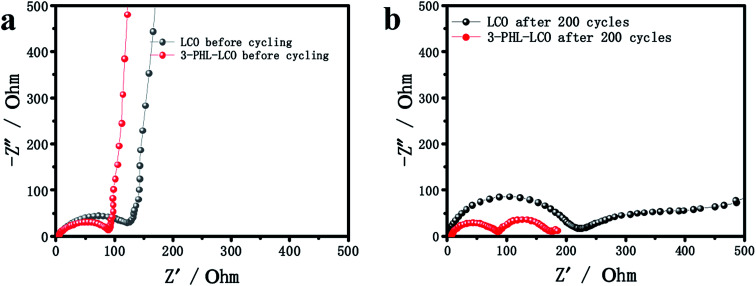
Impedance plots of uncoated LCO and 3-PHL-LCO (a) before and (b) after 200 cycles.

To gain more insights into the effect of the PHL coating layer on the electrochemical behavior, the AC impedance spectra of the bare LCO and 3-PHL-LCO at the discharge state before and after 200 cycles was analyzed as shown in Fig. 7. As is known to all, the semicircle in the high frequency region is attributed to the interface resistance of the surface membrane on the electrode active particles, and the semicircle in the medium-to-low frequency region is ascribed to the charge transfer resistance between the electrode active particles and LE.^36–38^ In Fig. 7a, the interfacial resistance of the pristine LCO is higher than that of the 3-PHL-LCO before cycles. The reason for the reduced impedance may be the high ion conductivity of PHL GPE and the integrated structure of 3-PHL-LCO. Moreover, both the interfacial resistance and charge transfer resistance of the pristine LCO are remarkably higher than that of the 3-PHL-LCO after the 200th cycle (Fig. 7b). This indicates that the adverse interfacial reaction between the charged LCO surface and LE may result in the sharp capacity decaying of the original LCO (Fig. 5c). Overall, the polarization resistance (*R*_p_) of both samples increases with cycles going on, but the increase can be effectively inhibited by the PHL coating layer. The reduction of side reactions on the surface of 3-PHL-LCO may be due to the fact that PHL GPE with anti-oxidation ability can directly prevent side reactions from occurring on the surface of LCO and thereby reduce the impedance. During electrochemical cycling, the PHL electrolyte membrane is similar to a solid electrolyte interface (SEI) membrane, completely covering the surface of bare LCO particles and electrode (Fig. 6c and d), while there is no obvious SEI on the bare LCO. This membrane can be well preserved at the charge cut-off voltage of 4.6 V, preventing side reactions between LCO particles and LE. Therefore, the AC impedance results are well consistent with the above long-term cycling performance of all the samples.

**Fig. 8:**
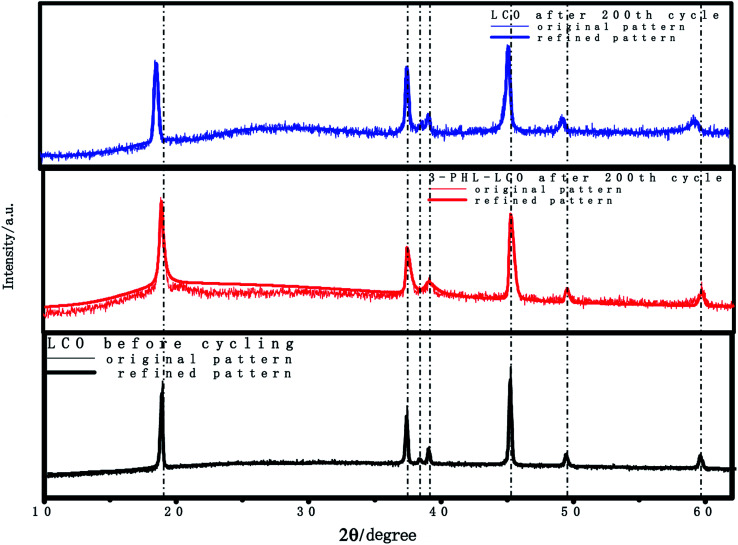
XRD patterns of LCO and 3-PHL-LCO after 200 cycles at charge cut-off voltage of 4.6 V.

According to previous reports,^39–41^ the structural stability and thermal stability of LCO can be disrupted, and there will be numerous side reactions with the charge cutoff voltage increasing, especially at the charge cutoff voltage of 4.6 V. The XRF was used to quantitatively analyze cobalt ions in the electrolyte of cycled cathodes after 200 cycles. The statistical results of quantitative elemental analysis are presented in Table 2. The content of dissolved cobalt ions from the cycled 3-PHL-LCO after 200 cycles is only 0.01% of the total active material mass, compared with 0.083% of cycled uncoated LCO. The large difference between the cycled cathodes with and without PHL membrane that demonstrates the coating can extend the cycling and well protect the LCO surface from Co^3+^ dissolution at the high charging voltage of 4.6 V. Furthermore, Fig. 8 shows the XRD patterns for coated and uncoated LCO cathodes after 200 cycles. Though the XRD refinement was used, no difference was found in the crystal structure (*R*-3*m*) and parameters (*a* = 2.81, *b* = 2.81, *c* = 14.05) of LCO and 3-PHL-LCO, but it is clearly seen that there is a obvious difference in the peak position with and without the PHL coating. The peak position of the 3-PHL-LCO cathode is more closed to that of the fresh LCO cathode. Whereas, the diffraction peaks of the cycled bare LCO cathode shift to the left. This may reflect that the bare LCO has suffered more severe structural damage than the 3-PHL-LCO at the high charge cut-off voltage of 4.6 V as a result of large capacity attenuation and low capacity retention. Besides, the thermal stability of the cathode material was characterized by cycling at 50 °C and 1C under charging voltage of 4.6 V. In Fig. S2,† 3-PHL-LCO presents a high cycle retention rate of 80.01%, while the LCO is only 61.25%. It shows that the thermal stability of LCO can be significantly improved by PHL coating.

**Table 2:** XRF results of cobalt ion content in the electrolyte after 200 cycles

Sample	Concentration/mg L^−1^	Tolerance/mg L^−1^	Active mass loss percentage/%
LCO	1.791	0.136	0.083 ± 0.006
3-PHL-LCO	0.215	0.002	0.010 ± 0.000

## 4. Conclusions

In this study, a facile and direct surface modification of preformed LCO cathode by the P(VDF-HFP)/LiTFSI (PHL) coating layer with high electrochemical stability prepared through the solvent-casting method. Then the surface and cross-sectional morphology of the cathode have been deeply analyzed by SEM and EDS measurements. The distinctive features of the LCO with PHL coating layer (∼3 μm) were the highly-continuous and compact surface coverage and penetration of the bulk of LCO to form an integrated electrode. This unique structure of the 3-PHL-LCO can effectively improve the high-potential electrochemical behaviors and alleviate the side reactions between the delithiated LCO and liquid electrolyte at 4.6 V high potential. The AC impedance, XRD and XRF measurements after long cycling proved that the integrated PHL coating layer with ion-conductive protection played a key role in effectively suppressing undesired interfacial side reactions and stabilizing the structure of LCO and also significantly inhibiting the dissolution of Co^3+^. In addition, the SEM image of coated and non-coated LCO after long cycling was contrasted, the bare LCO was heavily cracked. However, the degradation of the 3-PHL-LCO cathode was far less. A continuous and compact thin PHL coating layer was preserved during long cycling, impeding crack initiation or growth.

The present work underlines that the PHL-based surface modification performed at low temperature is a promising alternative to inorganic material coatings for application in high-voltage lithium-ion batteries. In future studies, the PHL-coated LCO charged to higher cut-off charge voltages (>4.6 V) will be examined and the structures and components of PHL coating layers will be further optimized.

## Conflicts of interest

There are no conflicts of interest to declare.

## Supplementary Material

RA-010-D0RA04023A-s001
