# Supplementary material for: Direct surface coating of high voltage LiCoO2 cathode with P(VDF-HFP) based gel polymer electrolyte
Source: RSC Adv. 2020 Jun 26;10(41):24533–41. doi: 10.1039/d0ra04023a (PMC9055184; doi:10.1039/d0ra04023a)
Supplement: RA-010-D0RA04023A-s001 [file RA-010-D0RA04023A-s001.pdf]

Electronic Supplementary Material (ESI) for RSC Advances.
This journal is © The Royal Society of Chemistry 2020

Supporting Information

Direct surface coating of high voltage LiCoO2 cathode with P(VDF-HFP) based gel polymer electrolyte

Huiling Chen, Yuehua Wen*, Yue Wang, Songtong Zhang, Pengcheng Zhao, Hai Ming, Gaoping Cao, Jingyi Qiu*

(Beijing Key Laboratory of Advanced Chemical Energy Storage Technology and Materials, Research Institute of Chemical Defense, Beijing 100191, China. E-mail: wen_yuehua@126.com; qiujingyi1202@163.com)

1. Variation of dQ/dV curves

In Fig. S1, it can be clearly seen that the change of the discharge platform and the degree of polarization as the number of cycles increases. And the 3-PHL-LCO with well maintained capacity exhibits a small increase in polarization, but the reaction polarization of original LCO increases significantly, which is consistent with the result of charge-discharge curves of bare LCO and 3-PHL-LCO (Fig. 5d and e).

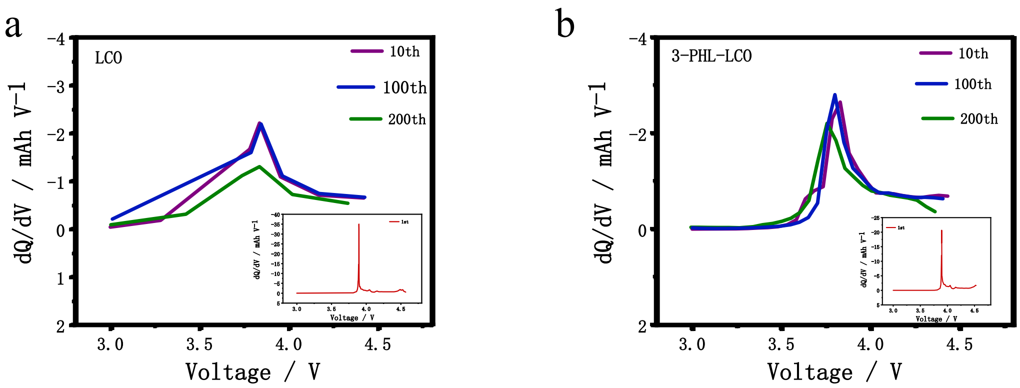

Fig. S1. Variation of dQ/dV curves at 1st, 10th, 100th, 200th cycle of (a) Bare LCO and (b) 3-PHL-LCO.

2.Characterization of thermal stability of 3-PHL-LCO under charging voltage of 4.6 V

The thermal stability of the cathode material was characterized by cycling at 50 ℃ and 1 C under charging voltage of 4.6 V. In Fig. S2, 3-PHL-LCO presents a high cycle retention rate of 80.01%, while the LCO is only 61.25%. It shows that the thermal stability of LCO can be significantly improved by PHL coating.

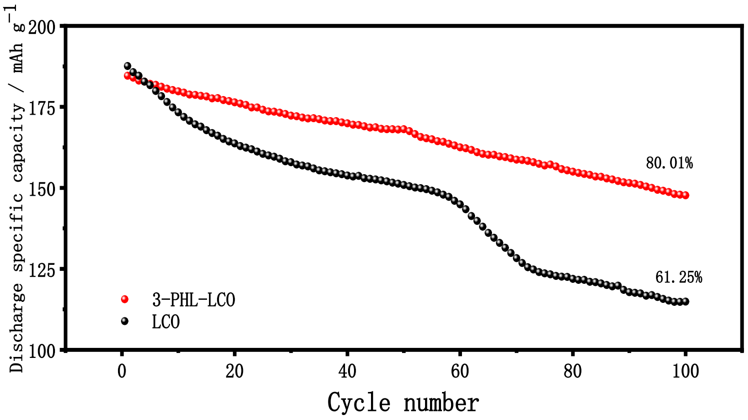

Fig. S2. Long cycle performance of bare LCO and 3-PHL-LCO at 50 ℃ between 3.0 and 4.6 V.
